# Fruits hidden by green: an improved YOLOV8n for detection of young citrus in lush citrus trees

**DOI:** 10.3389/fpls.2024.1375118

**Published:** 2024-04-10

**Authors:** Gao Ang, Tian Zhiwei, Ma Wei, Song Yuepeng, Ren Longlong, Feng Yuliang, Qian Jianping, Xu Lijia

**Affiliations:** ^1^ College of Mechanical and Electronic Engineering, Shandong Agricultural University, Tai’an, Shandong, China; ^2^ Institute of Urban Agriculture, Chinese Academy of Agricultural Sciences, Chengdu, China; ^3^ College of Engineering, China Agricultural University, Beijing, China; ^4^ Institute of Agricultural Resources and Regional Planning, Chinese Academy of Agricultural Sciences, Beijing, China; ^5^ College of Mechanical and Electrical Engineering, Sichuan Agriculture University, Ya’an, China

**Keywords:** YOLO V8, young citrus fruit, deep learning, target detection, lightweight network

## Abstract

In order to address the challenges of inefficiency and insufficient accuracy in the manual identification of young citrus fruits during thinning processes, this study proposes a detection methodology using the you only look once for complex backgrounds of young citrus fruits (YCCB-YOLO) approach. The method first constructs a dataset containing images of young citrus fruits in a real orchard environment. To improve the detection accuracy while maintaining the computational efficiency, the study reconstructs the detection head and backbone network using pointwise convolution (PWonv) lightweight network, which reduces the complexity of the model without affecting the performance. In addition, the ability of the model to accurately detect young citrus fruits in complex backgrounds is enhanced by integrating the fusion attention mechanism. Meanwhile, the simplified spatial pyramid pooling fast-large kernel separated attention (SimSPPF-LSKA) feature pyramid was introduced to further enhance the multi-feature extraction capability of the model. Finally, the Adam optimization function was used to strengthen the nonlinear representation and feature extraction ability of the model. The experimental results show that the model achieves 91.79% precision (P), 92.75% recall (R), and 97.32% mean average precision (mAP)on the test set, which were improved by 1.33%, 2.24%, and 1.73%, respectively, compared with the original model, and the size of the model is only 5.4 MB. This study could meet the performance requirements for citrus fruit identification, which provides technical support for fruit thinning.

## 1 Introduction

Citrus fruits are extensively cultivated globally, with countries such as China, Brazil, India, the United States, and Mexico ranking among the top nations in terms of citrus cultivation area (Lyu et al., 2022). This prominence establishes citrus as a crucial cash crop and a significant contributor to the agricultural economy. Effective management during the young fruit stage of citrus plays a pivotal role in influencing fruit growth, development, and overall quality. Consequently, adjusting the tree load during this period becomes essential to promote the development and enhance the quality of young fruits. The current practice of citrus young fruit thinning primarily relies on manual interventions, resulting in drawbacks such as low efficiency, imprecision, and higher costs. Leveraging machine learning and artificial intelligence technologies for intelligent fruit thinning holds the potential to improve work efficiency, ensure superior fruit quality, and concurrently reduce production costs. Therefore, intelligent fruit thinning stands as a promising avenue with distinct advantages and developmental prospects.

Automated identification and detection of young citrus fruits constitute a crucial stage in intelligent fruit thinning. The swift advancement of deep learning and computer vision technologies in recent years has opened new avenues for addressing this challenge. Specifically, the YOLO (You Only Look Once) series of first-order target detection algorithms have demonstrated noteworthy success in image processing and computer vision tasks. Furthermore, these algorithms have found extensive applications in the agricultural domain (Akkem et al., 2023; Farjon et al., 2023; Wu et al., 2023). Jintao Feng et al. (2023) proposed a YOLOX-based real-time multi-type surface defect detection algorithm (MSDD-YOLOX) for oranges in order to achieve real-time detection of orange surface defects on an orange sorter. The algorithm improves the detection of scars at different scales by introducing necking network residual connections and cascading of necking networks. To address the problem of missed detection in texture-based defects and to improve the regression of the predicted bounding box, focus loss and CIoU were used in the algorithm. The results show that MSDD-YOLOX achieves F1 values of 88.3%, 80.4% and 92.7% for the detection of deformities, scars, and lesions, respectively, with an overall detection F1 value of 90.8% (Feng et al., 2023). Chaojun Hou et al. (2022) proposed a new method to detect and localize ripe citrus using You Only Look Once (YOLO) v5s with improved binocular vision. In order to recover the missing depth information due to random overlapping of background participants, Cr-Cb chromaticity mapping, Otsu thresholding algorithm, and morphological processing are sequentially used to extract the complete shape of the citrus, and a kriging method was applied to obtain the optimal linear unbiased estimator of the missing depth values. Finally, the spatial position and attitude information of the citrus were obtained based on the camera imaging model and the geometrical features of the citrus. The experimental results showed that the recall of citrus detection under non-uniform lighting conditions, weak lighting and lighting conditions were 99.55%, 98.47% and 98.48% (Hou et al., 2022). Sadaf Zeeshan et al. (2023) proposed a deep learning convolutional neural network model for orange fruit detection using a generic real-time dataset for detecting oranges in complex dynamic environments. A Keras sequential convolutional neural network model with convolutional layer activation functions, maximum pooling and layers fully connected was developed. Images acquired from an orchard using a Kinect RGB-D camera were used to evaluate the model. The accuracy of the proposed CNN model was 93.8%, precision was 98%, recall was 94.8% and F1 score was 96.5% (Zeeshan et al., 2023). In citrus detection, scholars have done research on citrus surface defects, spatial localization of citrus position and citrus quantity detection using deep learning techniques, however, little research has been done in the detection of young citrus fruits.

Despite remarkable progress in target detection using deep learning, challenges remain in detecting citrus fruit in complex orchard backgrounds, varying lighting conditions, occlusions and fruit size differences. To address these issues, a novel YCCB-YOLO model specifically tailored for citrus fruit detection was proposed. This model aims to provide effective technical support for automated orchard management, enabling a more efficient and intelligent approach.

The study was structured as follows: an introduction outlining the research background, importance, existing challenges and the novelty of our approach was provided. The methods section details the construction of the comprehensive dataset representing real citrus young fruit images, and the design and implementation of the YCCB-YOLO model. Experimental results covering the comparison of the proposed method with existing techniques are then presented to demonstrate its effectiveness. Finally, the study concludes with a summary of our main contributions, findings and directions for future research.

The main contributions of this study are as follows:

Model innovation: We introduce the YCCB-YOLO model, which achieves accurate citrus young fruit detection in complex backgrounds through optimized network architecture and integrated attention mechanisms.

Dataset development: A comprehensive dataset of citrus fruit images in authentic orchard environments will be established, providing a rich resource for model training and evaluation.

Performance enhancement: By incorporating the SimSPPF-LSKA feature pyramid and using the Adam optimization function, we significantly improve the detection accuracy and computational efficiency of our model.

Real-time application potential: The proposed method exhibits compact model size and high computational efficiency, making it suitable for real-time citrus fruit detection applications.

## 2 Experiments and methods

### 2.1 Image acquisition and pre-processing

**Figure 1:**
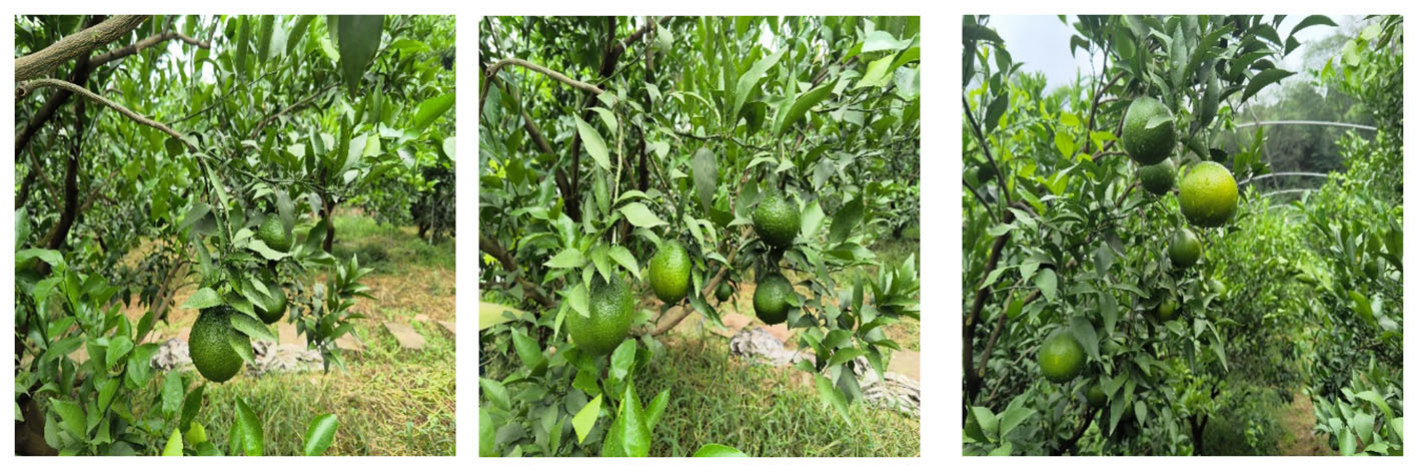
Images of young citrus fruit parts at different angles.

In this study, the collection work of citrus young fruit image data was carried out and the citrus young fruit dataset was established. The collection site was selected in citrus plantation in Huanglongxi Town, Shuangliu District, Chengdu City, Sichuan Province (longitude 30°19′21.84″, latitude 103°57′48.57″), while the collection tool was a Redmi K60 ultra mobile phone (Sony IMX596 camera). During the filming process, three different time periods, namely, morning, noon and evening, were selected to simulate different lighting conditions during the day, so as to gain a more comprehensive understanding of its morphology and characteristics. During the filming process, single pictures and videos were taken, after obtaining the image data, the key frames of the videos were selected and converted into pictures. By screening and labelling the images, blurred, overexposed as well as duplicated hard-to-label images were eliminated, and clear, high-quality and representative images were retained. Finally, 1400 valid images of young citrus fruits were obtained, some of which are shown in **Figure 1**. The young citrus fruit targets in the images were labelled using Labelimg image annotation tool and the images and labelled data were stored in PASCAL VOC format. After completing the labelling, the dataset was divided into training, testing and validation sets in the ratio of 7:2:1 (Gao et al., 2022).

### 2.2 YCCB-YOLO detection model

**Figure 2:**
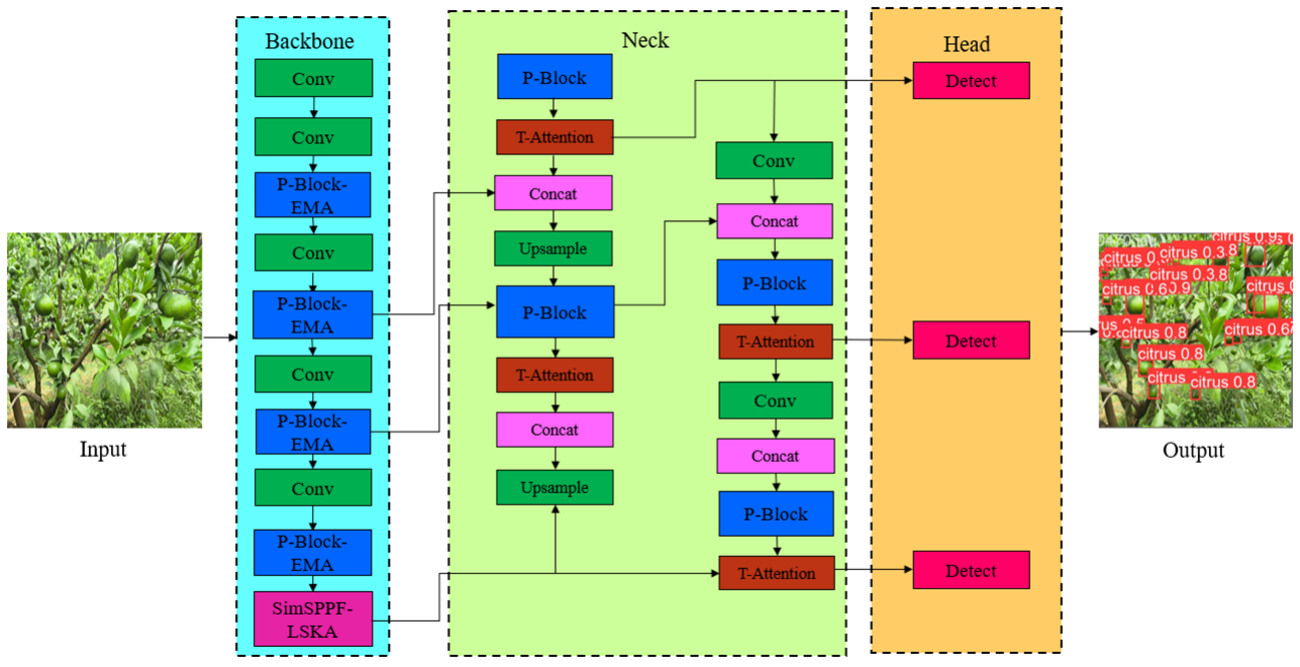
Structure of citrus young fruit detection model.

The YCCB -YOLO citrus young fruit detection model adopts the YOLO v8n model as the basic architecture, which is composed of backbone, head and Detect respectively. In order to further improve the detection accuracy and lightweight of the model, backbone and head were improved by light weighting, fusing the attention module, adopting the fast inter-pyramid pooling method, as well as optimally selecting the Adam optimizer, finally the structure of the citrus young fruit detection model was as shown in **Figure 2**.

**Figure 3:**
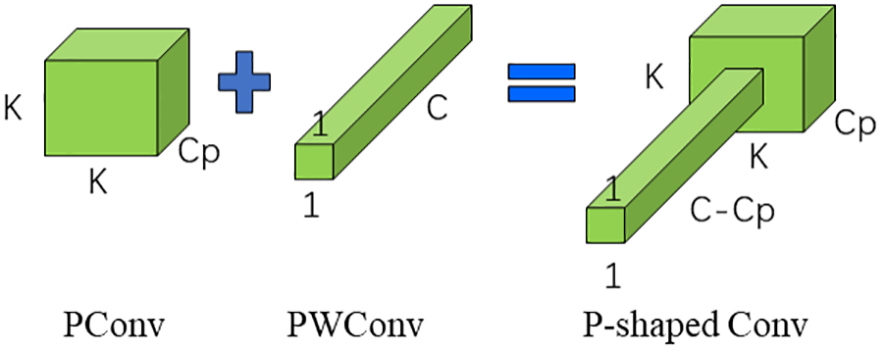
Schematic diagram of P-shaped Conv convolution.

#### 2.2.1 Lightweight backbone, head designs

The lightweight backbone, head design provides more advantages for the citrus young fruit detection model in embedded device applications. The YOLO series backbone network mainly performs image feature extraction, using a convolution kernel to perform convolution operation with the input image after receiving the input image data in order to capture the local features of the image, while an upsampling operation will be added in the post-processing stage to perform feature fusion (Jiang et al., 2024).

The detection head mainly performs target recognition based on the information of the feature map and outputs the category and location information of the target. The network firstly performs convolution operation on the feature map output from backbone to extract feature information, then it uses fully connected layer to classify and regress the feature information. Fully connected layer will classify each pixel location, determine whether there exists a target at that location, and output the location and category information of the target (Xiao et al., 2023).

It could be seen that the convolutional layer conv plays a crucial role in YOLO, and different convolutional kernels, step sizes, activation functions, and network structures make up different convolutional variants (Basha et al., 2019). These variants target specific tasks and data to further improve the performance of the convolution into performance and adaptability. The final application scenario of this study was an orchard with a complex environment, which has higher requirements for model accuracy, size and detection speed, so a lightweight convolutional network structure was used for head and backbone.

Deep separable convolution reduces the number of parameters and computational complexity in the model by splitting the convolution operation into two parts, deep convolution and pointwise convolution, thus reducing the model size and runtime (Hong et al., 2021). Therefore depth separable convolution was commonly used in many lightweight models, but the frequent memory access of this convolution was still a problem to be solved, PConv used the redundancy of feature maps to further optimize the cost to solve the problem of frequent memory access nicely (Chen et al., 2023).

PConv unique in that it will select the first or last consecutive cp channel as a representative of the overall feature map to be computed when performing consecutive or standard memory accesses (Chen et al., 2023). The FLOP was calculated as follows in Equations 1–3:


(1) $$\text{h}\times \text{w}\times k^{2}\times c_{p}^{2}$$

(2) $$\text{h}\times \text{w}\times 2c_{p}+k^{2}\times c_{p}^{2}$$

When the regular convolution r = 1/4, which is only 1/4 of its.


(3) $$c_{1}\times \frac{h}{4}\times \frac{w}{4}$$

At the same time, in order to be able to use the information of all channels, a pointwise convolution (PWonv) was attached to the PConv, which finally shows the schematic of the convolution in **Figure 3**.

**Figure 4:**
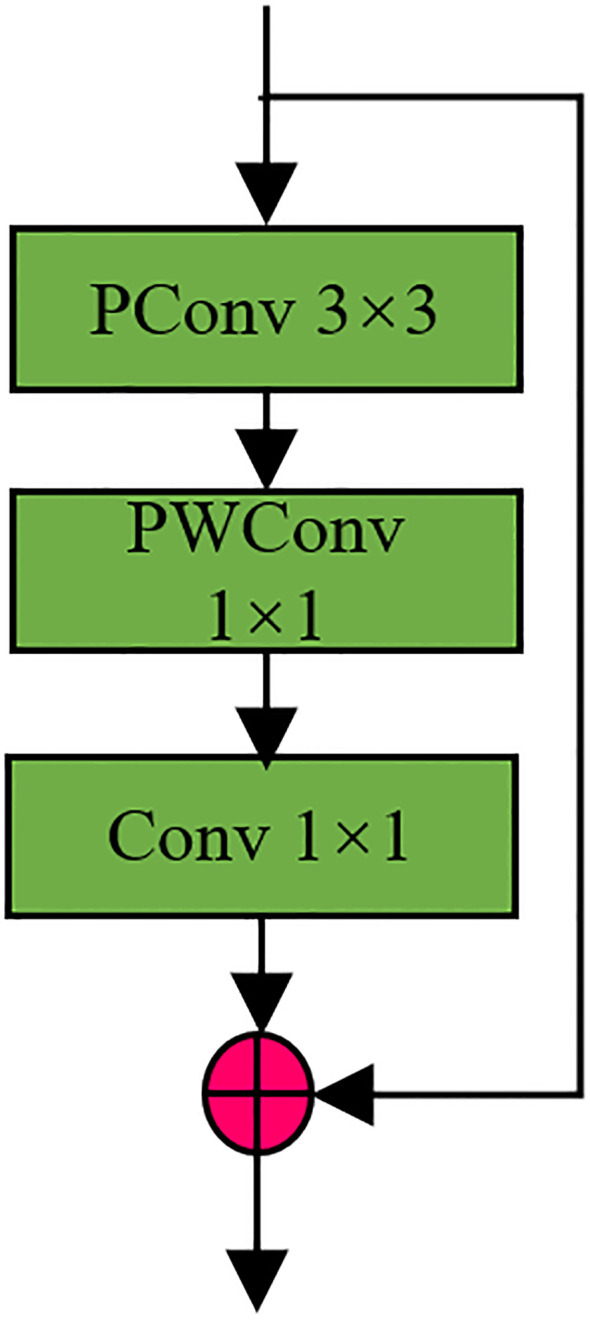
Schematic diagram of P-Block module.

**Figure 5:**
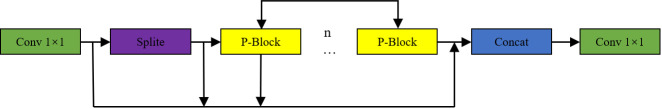
Schematic diagram of P-Block module fusion C2F.

Based on PConv convolution this paper redesigns the Bottleneck module in C2F in YOLOV8, named P-Block, and its structure diagram as shown in **Figure 4** below, P-Block adds a layer of ordinary 1*1 convolution and residual operation on the basis of P-shaped Conv to better optimize the whole module. In order to backbone and head lightweight, all the c2f modules in YOLOV8 were replaced with P-Block (Bao et al., 2023), and the P-Block module fused C2F as shown in **Figure 5**.

#### 2.2.2 Attentional mechanisms of fusion

Attention mechanism is a method in deep learning that mimics human attention allocation, helping neural networks capture long-distance dependencies in input sequences more effectively by automatically learning to weight and focus on key information (Wang and Jiao, 2022). In deep learning target detection techniques, the attention mechanism automatically identifies and focuses on key regions in the input image, thus improving the accuracy and robustness of target detection with reduced computational resource consumption. By fusing different attention mechanisms together, the advantages of each mechanism as well as their respective shortcomings could be fully utilized. Because different attention mechanisms focus on problems from different perspectives and in different ways, fusing them together could complement each other and capture the characteristics of the input data in a more comprehensive way. Secondly, integrating multiple attention mechanisms improves the diversity of the model, diverse information processing methods help to improve the robustness and generalization ability of the model. Finally, fusing multiple attention mechanisms has flexibility in that it could be flexibly combined according to specific tasks and data characteristics to adapt to different application scenarios (Wan et al., 2023). Therefore, in this paper, a fused attention mechanism was added to the citrus young fruit detection model to improve the detection accuracy of the model.

**Figure 6:**
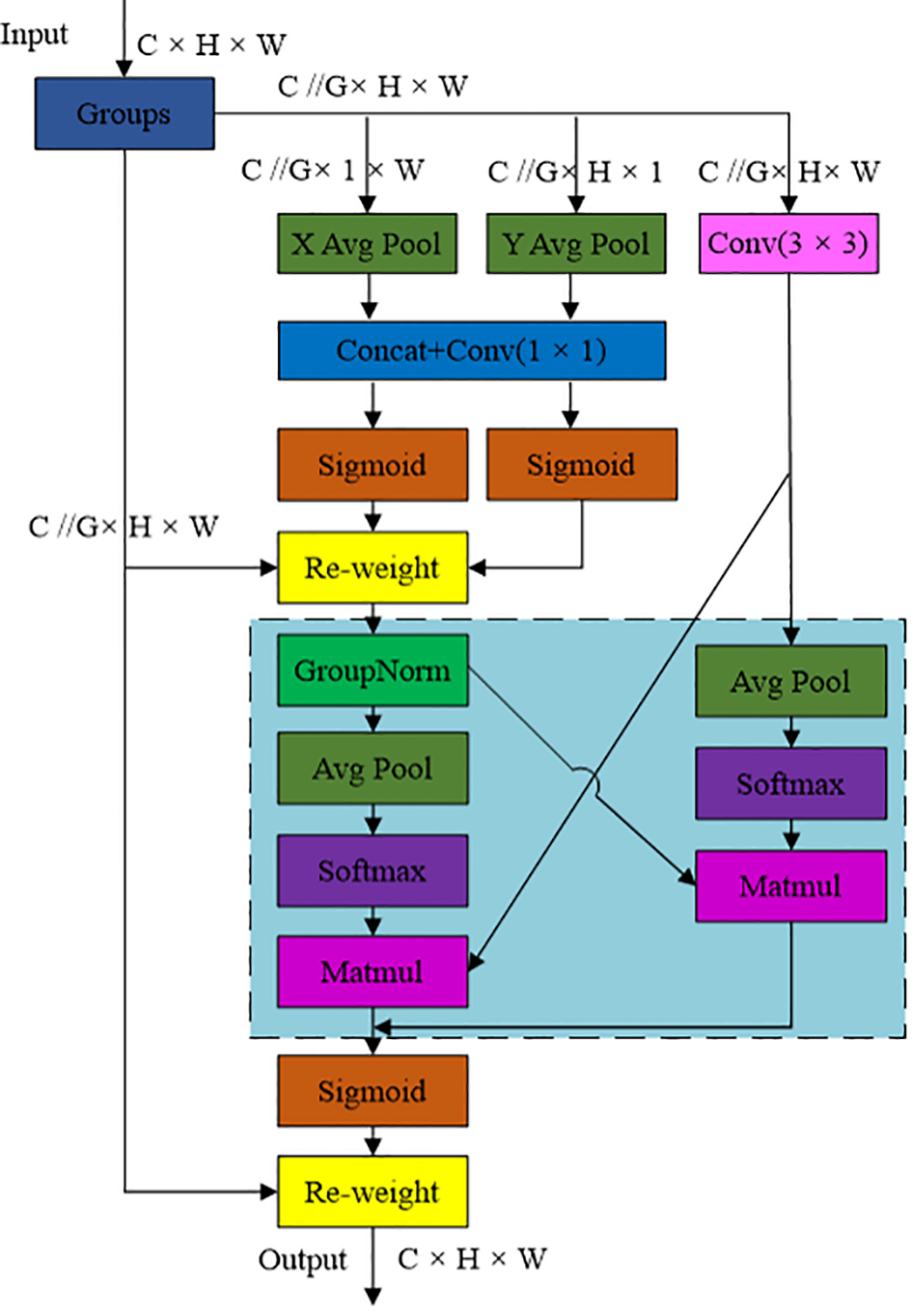
Schematic diagram of the efficient multi-scale attention module of EMA.

Efficient multi-scale attention (EMA) is an efficient multi-scale attention mechanism that can effectively solve the accuracy degradation problem caused by channel dimensionality reduction in model construction. **Figure 6** shows the structure diagram of the EMA module. From the figure, it could be seen that the module transforms some channels into batch dimensions, groups channel dimensions into multiple subfeatures, encodes global information to recalibrate the weights of parallel channels, then aggregates feature outputs across dimensions, while ensuring that the information of each channel was focused without increasing the computational complexity (Ouyang et al., 2023).

**Figure 7:**
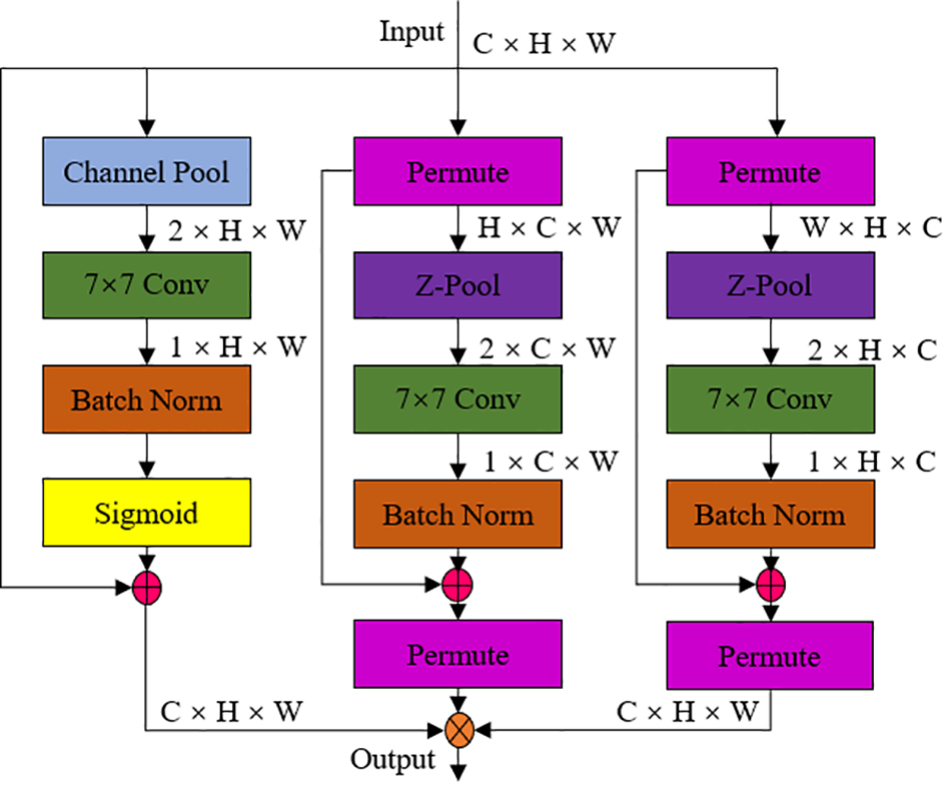
Schematic diagram of Triplet Attention ternary attention mechanism module.

The Triplet Attention ternary attention mechanism implements the interaction of information between the channel dimension and the spatial dimension in an almost parameter-free way constructing the channel attention and spatial attention. The structural sketch of the Triplet Attention module shows in **Figure 7**, from which it could be seen that the module captures the information of the interaction of the quartal dimensions through three parallel branch structures. The first branch captures the interaction features between the channel dimension and the spatial dimension. It interacts the channel dimension in the input tensor with the spatial dimension to extract the channel features at different spatial locations. The second branch interacts the channel dimension with another spatial dimension to capture the interaction between channel features at different spatial locations. The third branch was used to build spatial attention. It generates a global representation of the channel by aggregating the features of the channel dimension to capture the interactions between different channels, thus improving the feature extraction capability of the module in different dimensions (Lau et al., 2024).

**Figure 8:**
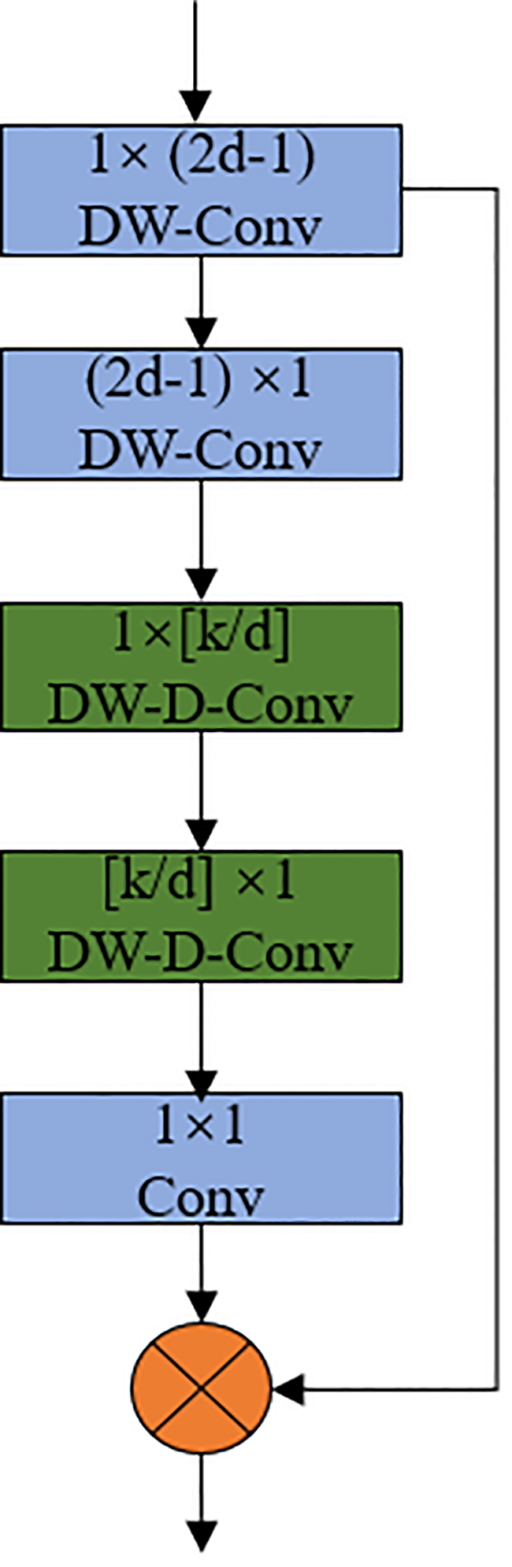
LSKA Large Separable Kernel Attention Module.

The Large Separable Kernel Attention (LSKA) module solves the problem of large convolutional kernels showing a quadratic increase in computation and memory for deep convolutional layers. The structure of the LSKA module shown in **Figure 8**. As shown in the structure diagram, the module decomposes the 2D convolutional kernels of the deep convolutional layer into cascaded horizontal and vertical 1-D kernels to reduce the computational complexity (Zhichao et al).

In this paper, the design of the citrus young fruit recognition model focuses on the lightweight of the detection model, but the lightweight will inevitably bring the loss of detection accuracy. Therefore, a fused attention mechanism was adopted to improve the detection accuracy of the model. The Triplet Attention ternary attention mechanism was used in the detection head, the EMA attention mechanism was adopted in the detection backbone, and the LSKA attention mechanism was adopted in the feature pyramid, and the location map of their added attention mechanisms as shown in **Figure 2**.

#### 2.2.3 Feature pyramid networks

**Figure 9:**
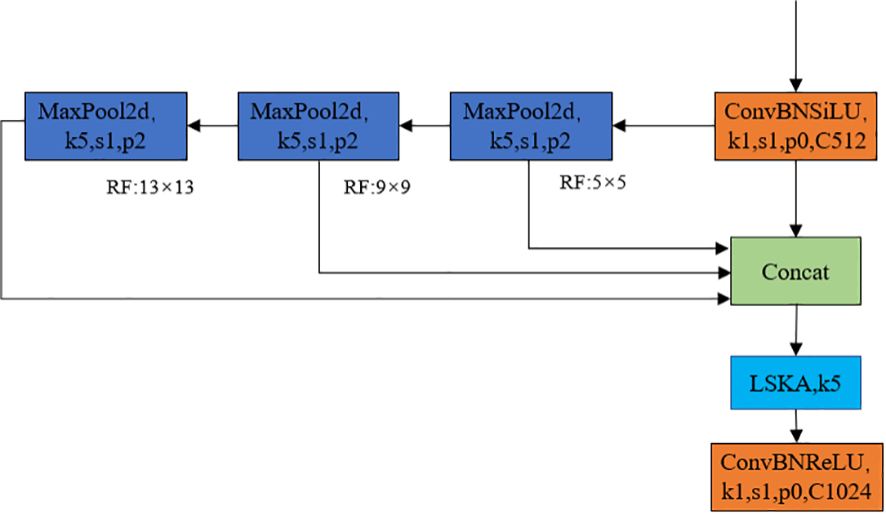
Schematic diagram of SimSPPF- LSKA feature pyramid structure.

The feature pyramid network can fuse feature information from different scales and reduce the loss of small targets (Deng et al., 2021; Huang et al., 2021). The SPPF feature pyramid structure has been adopted in yolov8. It reduces the amount of computation by three consecutive maximum pooling, the convolution kernel unified as 5*5, finally concat the results before pooling and after each pooling, meanwhile ensures the effect of multi-scale fusion achieves the fusion of local features and global features at the level of featherMap (Tang et al., 2023). In the citrus young fruit dataset there was some noise and interference, so in order to improve the robustness of the model, the SimSPPF structure (Hu and Zhu, 2023; Wang et al., 2023) was introduced and the large kernel separated attention (LSKA) was used in the architecture, which was called the feature pyramid structure as SimSPPF- LSKA, and the structure as shown in **Figure 9**.

As shown in **Figure 9**, SimSPPF-LSKA has been optimized compared to SPPF in the choice of activation function, with SPPF using SiLU (Sigmoid Linear Unit) as the activation function, while SimSPPF uses ReLU (Rectified Linear Unit), this change improves the speed of each module, making SimSPPF more efficient compared to SPPF. In addition, the LSKA attention module with a 5 × 5 convolutional kernel has been incorporated after three consecutive maximum pooling. The LSKA module enables the model to accurately capture important feature information at different scales by combining the local and global attention mechanisms, thus improving the quality of feature representations to enhance the performance of the model.

## 3 Results and discussion

### 3.1 Test environment and parameter configurations

The hardware environment for this test is Lenovo R9000P2021H with AMD Ryzen 7 5800H processor, 16GB of RAM on board, RTX3060 Laptop GPU graphics card, and Windows 10 Home Edition 2021 system.

A column of key parameters were carefully chosen for model training as shown in **Table 1**, as shown in **Table 1** the input image size as 640×640, batch size as 16, initial learning rate as 0.001, etc., these parameters were set to ensure efficient training and validation of the model to maximize the performance and accuracy of the model.

**Table 1:** Table of model training parameters.

Parameter name	Configuration value
Input image size	640×640
Initial learning rate	0.001
Batch size	16
Number of training epochs	100
Optimizer	SGD/Adam/AdamW
Momentum	0.937

### 3.2 Evaluative indicators

In this paper, the target detection model has been comprehensively evaluated using evaluation metrics such as precision rate, recall rate, mAP50 and test time per image. These evaluation metrics comprehensively measure the performance of the model in terms of classification accuracy, localization accuracy and operational efficiency, thus the comprehensive evaluation of these metrics provides important guidance for model optimization (Guo et al., 2023; Huang et al., 2023). Precision, Recall, AP, and mAP calculation formulas follow as shown in Equations 4–7 below.


(4) $$Precision=\frac{T_{P}}{T_{P}+F_{P}}\times 100\%\;\;\;\;\;\;\;\;\;\;\;\;\;\;\;\;\;\;\;\;\;\;\;\;\;\;\;\;\;\;\;\;\;\;\;\;\;\;\;$$

(5) $$Recall=\frac{T_{P}}{T_{P}+F_{N}}\times 100\%\;\;\;\;\;\;\;\;\;\;\;\;\;\;\;\;\;\;\;\;\;\;\;\;\;\;\;\;\;\;\;\;\;\;\;\;\;\;$$

(6) $$AP=\int_{0}^{1}P\left(R\right)dR\;\;\;\;\;\;\;\;\;\;\;\;\;\;\;\;\;\;\;\;\;\;\;\;\;\;\;\;\;\;\;\;\;\;\;\;\;\;\;\;\;$$

(7) $$mAP=\frac{1}{K}\sum_{i=1}^{K}AP\left(i\right)$$

where: Precision-precision, Recall-recall rate, mAP-mean average precision

T_P_-number of correctly detected citrus fruit.

F_P_-Number of incorrectly detected young citrus fruit.

F_N_-number of missed citrus fruits.

AP- Area under the P and R curves.

### 3.3 Analysis of results

#### 3.3.1 Analysis of model training and validation process

**Figure 10:**
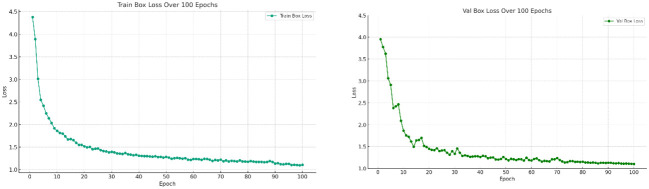
Loss map for model training and validation.

Deep learning models are known as “black boxes” due to their complex network structure and large number of parameters, whose internal reasoning process often remains opaque, posing challenges for model training and evaluation. In order to increase the interpretability of the model and prove the effectiveness of training and evaluation, loss function analysis was used in this study. As shown in **Figure 10**. The loss function values were monitored during the training process and the loss plots for training and validation were plotted. From **Figure 10** it can be seen that the model achieved proper convergence as the number of training rounds increased, therefore our model and training evaluation were valid.

#### 3.3.2 Analysis of result verification for lightweight P-Block

**Figure 11:**
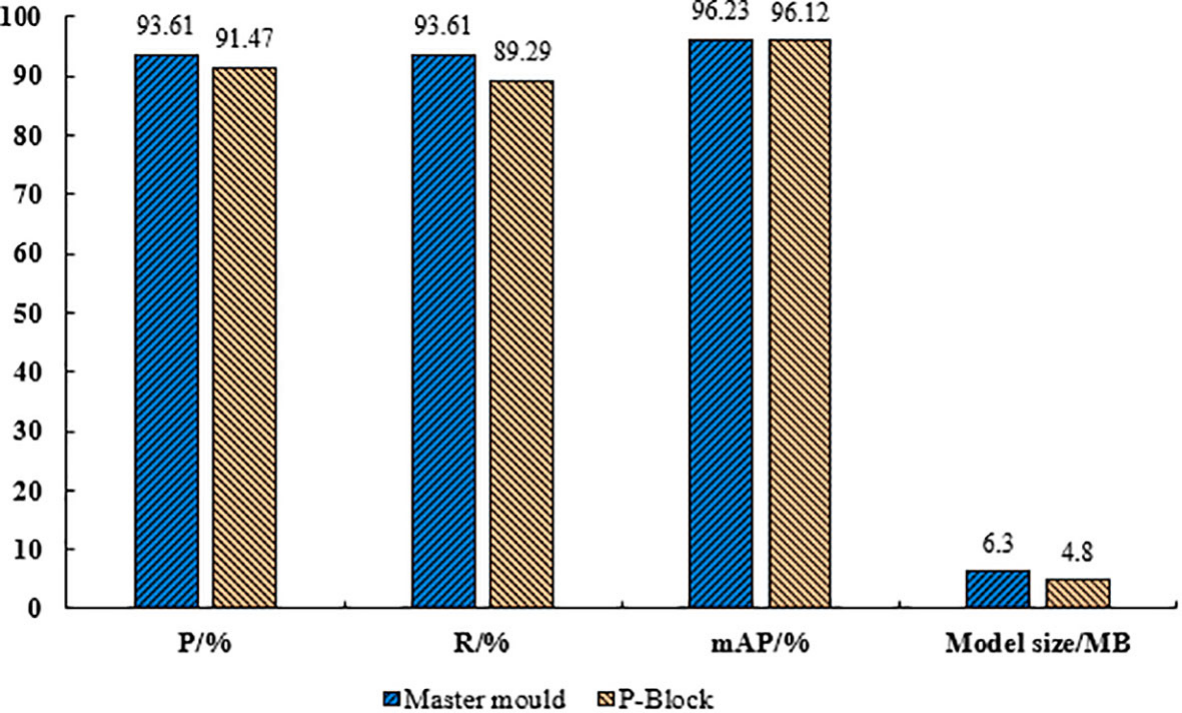
Comparison test results of lightweight P-Block with the original model.

In this paper, the model adopts P-Block module for light weighting, in order to verify the feasibility of the method the model with P-Block module and the original model are compared and analyzed for various evaluative metrics, the results as shown in **Figure 11**. As could be seen from **Figure 11**, the size of the model with P-Block in both the head and the backbone was 4.8 MB, which was 20.6% less compared to the original model, the precision was 91.47%, which was 2.14% less compared to the original model, the recall was 89.29%, which was 1.04% less compared to the original model, and the mAP was 96.12%, which was 0.11% less compared to the original model. The size of the model has been compressed after light weighting and at the same time there was a negative impact on the overall performance of the model.

#### 3.3.3 Result validation analysis of the attentional mechanism of fusion

The model in this paper fuses different types of attention modules at different locations, in order to verify the effectiveness of fusing multiple attention mechanisms, the model with fused attention mechanisms was tested in comparison with the original model, and the results were shown in **Table 2**. As shown in **Table 2**, the citrus young fruit detection model fused with multiple attention mechanisms improves in detection performance, compared with the model without added attention modules the precision improves by 0.24%, the recall improves by 1.67%, and the mAP improves by 0.12%, meanwhile, it was found that different parts of the addition of the attention modules have different effects, the addition of the T-Attention attention module to the head of the mAP improved by 0.02%, P decreased by 0.63%, and R decreased by 1.96% compared to fusing multiple attention modules. This proves that different attention modules in different locations have different performance in improving the detection indexes, while the same comparison finds that the size of the model increases by 0.1MB compared to that of the lightweight P-Block, which indicates that the fusion of multiple attention mechanisms will increase the parameters of the model and thus increase the size of the model while improving the performance of the model.

**Table 2:** Test results of the fused attention mechanism.

Model	Attention Module	P/%	R/%	mAP/%	Model Size/MB
YOLO V8- P-Block	×	91.47	89.29	96.12	4.8
YOLO V8- P-Block	Head + T-Attention	91.03	90.91	96.26	4.9
YOLO V8- P-Block	Backbone + EAM	90.76	89.45	96.17	4.9
YOLO V8- P-Block	Head + T-Attention、Backbone + EAM	91.71	90.96	96.24	4.9

#### 3.3.4 Result validation analysis of SimSPPF- LSKA feature pyramid

In this paper, SimSPPF- LSKA feature pyramid was used for the model, and large kernel separated convolutional attention was incrementally added while simplifying the feature pyramid to improve the ability of different scales feature fusion. In order to verify the feature fusion ability of SimSPPF- LSKA feature pyramid, a comparison test is carried out between the model of this paper using SimSPPF- LSKA feature pyramid and the model of this paper using SPPF, SPPF- LSKA, SimSPPF, and the results shown in **Table 3**. As indicated in **Table 3**, the LSKA attention module increases the model size by 0.5MB due to the addition of the large kernel separation convolution. in the precision comparison the pyramid is the highest for the SimSPPF model at 91.93%, the highest value of recall was 92.05% for the model using the SimSPPF- LSKA feature pyramid, and the highest value of mAP was 96.58%,which indicates that SimSPPF- LSKA feature pyramid improves the ability of feature fusion at different scales.

**Table 3:** Results of the improved feature pyramid structure.

Feature Pyramid	P/%	R/%	mAP/%	Model Size/MB
SPPF	91.71	90.96	96.24	4.9
SPPF- LSKA	91.84	91.74	96.33	5.4
SimSPPF	91.93	88.41	96.22	4.9
SimSPPF- LSKA	91.21	92.05	96.58	5.4

### 3.4 Discussion and analysis

#### 3.4.1 Discussion of the results of different light weighting models

Lightweight detection heads and detection backbones have been the focus of research, where lightweight target detection heads and backbone networks form the key to improving real-time target detection and reducing computational resource consumption. Therefore, this section focuses on the effects of different lightweight detection heads and backbone networks on the improved YOLOV8 series model. As shown in **Table 4**, P-Block is added into the detection head and backbone of YOLOV8, C3 lightweight detection backbone was adopted, fastestet was adopted as the detection backbone to compare and analyze with the model of this paper, respectively. As shown in **Table 5**, the detection performance of different combinations of detection head and detection backbone is different, when P-Block was used in both the detection head and the detection backbone, the model size of 4.8MB was the model with the lowest percentage of all models, while Map increased by 0.53% compared to the model with C3 module, and increased by 0.78% compared to the model with the detection head bit c2f and the backbone with fasternet, when considering the effects of detection performance and model size, the YOLO V8 model with P-Block achieves excellent performance.

**Table 4:** Results of the improved feature pyramid structure.

model	Head	Backbone	P/%	R/%	mAP/%	Model Size/MB
YOLO V8	C3	C3	90.46	90.51	95.59	5.2
YOLO V8	C2f	C2f	93.61	90.33	96.23	6.3
YOLO V8	P-Block	P-Block	91.47	89.29	96.12	4.8
YOLO V8	C2f	Fasternet	90.34	86.75	95.34	8.6

**Table 5:** Comparison of performance of different citrus recognition models.

Author	Data set	Model	P/%	R/%	mAP/%	Velocity
Hamzeh Mirhaji et al. (2021)	Self-built citrus trees	YOLO v4	91.23	92.8	90.8	/
Jintao Feng et al. (2023)	Self-built mature citrus	YOLO-DCA	/	/	96.98	Single sheet 5.9ms
Zhenhui Zheng et al. (2021)	Self-built green citrus	YOLO BP	86.00	91.00	91.55	18 frames (FPS)
Chaojun Hou et al. (2022)	Self-built mature citrus	Improved YOLO v5s	99.55	98.47	98.48	78.96 ms
Heqing Huang et al (Huang et al., 2021)	Self-built mature citrus	YOLO v5s-CBAM+ASFF+Purning	93.32	88.78	/	180 ms/frame
Sadaf Zeeshan et al. (2023)	Internet crawler	Orange-detection CNN	93.8	94.8	/	/
Aang Gao et al. (2022)	Self-built young citrus fruit	Improved YOLO v8 model	91.79	92.75	97.32	单张697.1ms

#### 3.4.2 Discussion of results for different optimization functions

**Figure 12:**
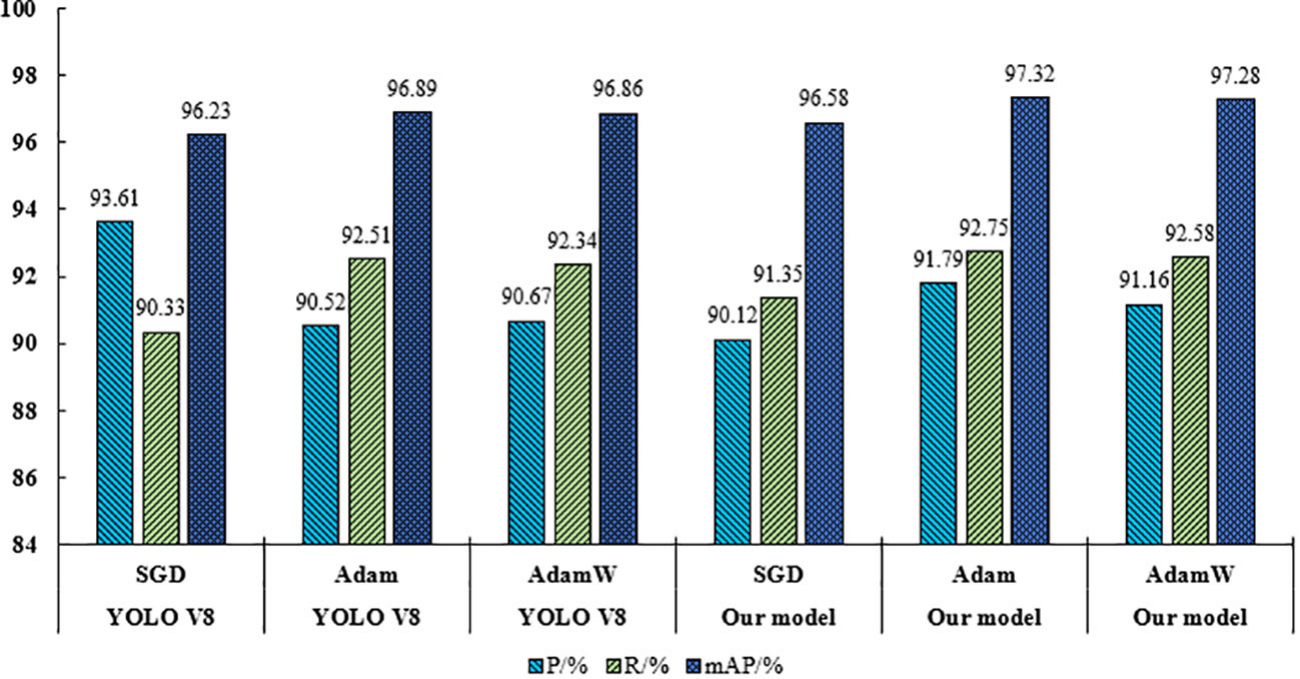
Comparison results of different optimization functions.

Different optimization methods have different expressive power in the YOLO series of models, this section compares the performance of SGD, Adam, and AdamW optimization functions in YOLO V8 with the citrus young fruit model (Loshchilov and Hutter, 2018; Jia et al., 2022), whose results are shown in **Figure 12**. From **Figure 12**, it could be seen that the model in this paper performs best when using Adam optimizer, and Adam optimizer significantly improves the performance of the model compared to other optimizers. Compared to the SGD optimizer, using the Adam optimizer can improve the P-value of this paper’s model by 1.67%, the R-value by 1.40%, and the mAP-value by 0.74%. Compared to the AdamW optimizer, using the Adam optimizer improves the P-value of this paper’s model by 0.63%, the R-value by 0.17%, and the mAP value by 0.04%. The model of this paper was compared with the YOLOv8 model in terms of optimizer selection. The P, R and mAP of this paper’s model were better than YOLOv8 when using the Adam optimizer. This paper’s model also performs well when using the AdamW optimizer, but is slightly inferior to YOLOv8.Therefore, the Adam optimizer was finally chosen for this paper’s model.

#### 3.4.3 Comparison of different citrus detection models

A variety of advanced deep learning models have been developed in the field of citrus recognition, where the accuracy and efficiency of citrus recognition has been greatly improved. In this paper, a variety of citrus detection models are selected for comparative analysis, as shown in **Table 5**. As shown in **Table 5**, different citrus recognition models differ in performance. It could be noted that the differences in the performance of different citrus recognition models were mainly related to the model structure, parameter settings and dataset quality, etc. Chaojun Hou et al. The improved YOLO v5s model with P of 99.55%, R of 98.47%, and mAP of 98.48% was the best performer among all the models. This paper’s model performs well in terms of accuracy, speed and mAP values and could meet the needs of different application scenarios.

#### 3.4.4 Advanced comparative analysis of benchmarking models

This section presents a detailed comparative analysis between the YCCB-YOLO model and current advanced benchmark models, including YOLO V5, YOLO V6, YOLO V7, and YOLO V8, as illustrated in **Table 6**. The YCCB-YOLO model demonstrates superior performance in the task of citrus young fruit detection, achieving a precision of 91.79%, a recall rate of 92.75%, and mAP of 97.32%. In comparison, YOLO V5 and YOLO V8 exhibit commendable precision rates of 90.81% and 90.46%, respectively, while YOLO V6 follows closely behind the YCCB-YOLO model in recall performance with a rate of 92.13%. YOLO V7 shows moderate performance across all metrics, particularly ranking lowest in mAP at 94.47%. The high efficiency and accuracy of the YCCB-YOLO model in detecting young citrus fruits underscore its advantages, while also highlighting the performance of other models in specific metrics. This analysis provides valuable insights for the future improvement and optimization of models.

**Table 6:** Comparison results with advanced benchmark models.

model	P/%	R/%	mAP/%
YOLO V5	90.81	88.04	95.24
YOLO V6	88.92	92.13	96.11
YOLO V7	89.43	91.18	94.47
YOLO V8	90.46	90.51	95.59
The model of this paper	91.79	92.75	97.32

#### 3.4.5 Portability of model field movement detection

The cost of hardware must be considered when building a citrus young fruit detection system, and the low price and good detection effect will be accepted by the orchard operators (Cubero et al., 2016). Therefore, the relatively inexpensive embedded devices were chosen for citrus young fruit detection, but the low-cost embedded devices have limited processing power to put forward better requirements for the model, so the test on the embedded devices was more valuable for practical applications. The system used in this paper mainly consists of a micro computer processor (Advantech AiMC-200J), camera, and monitor. The memory of the microcomputer processor with 2GB, the processor with Inter Celeron CPU J1900, the disk capacity of 128GB, the operating system micro Ubuntu 20.04.

**Figure 13:**
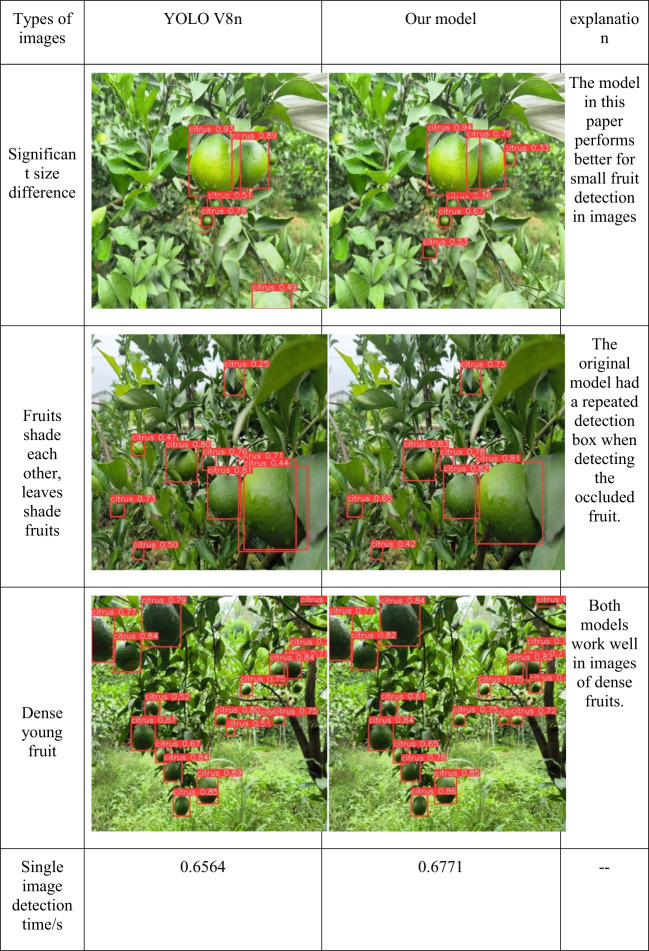
Testing of different models in the detection system.

Fifty images of young citrus fruits in the test set of this paper’s dataset were randomly selected for testing this paper’s model and YOLO V8n in this system, and the results are shown in **Figure 13**. The detection accuracy of this paper’s model was inferior to that of the YOLO V8n model in the images with sparse young fruits, but the V8n model showed leakage detection. In partially occluded images the detection frame of this paper’s model more accurately identifies the larger fruits at the front end, and the overall detection accuracy of this paper’s model was higher than that of the YOLO V8n model in images with dense young fruits. However, in the detection time of V8n model, the average detection time of a single sheet of 0.6564 seconds, and the average detection time of a single sheet of 0.6971 seconds of this paper’s model increased by 0.0207 seconds. Taken together this paper’s model was more advantageous in the face of dense young fruits and shaded young fruits.

**Figure 14:**
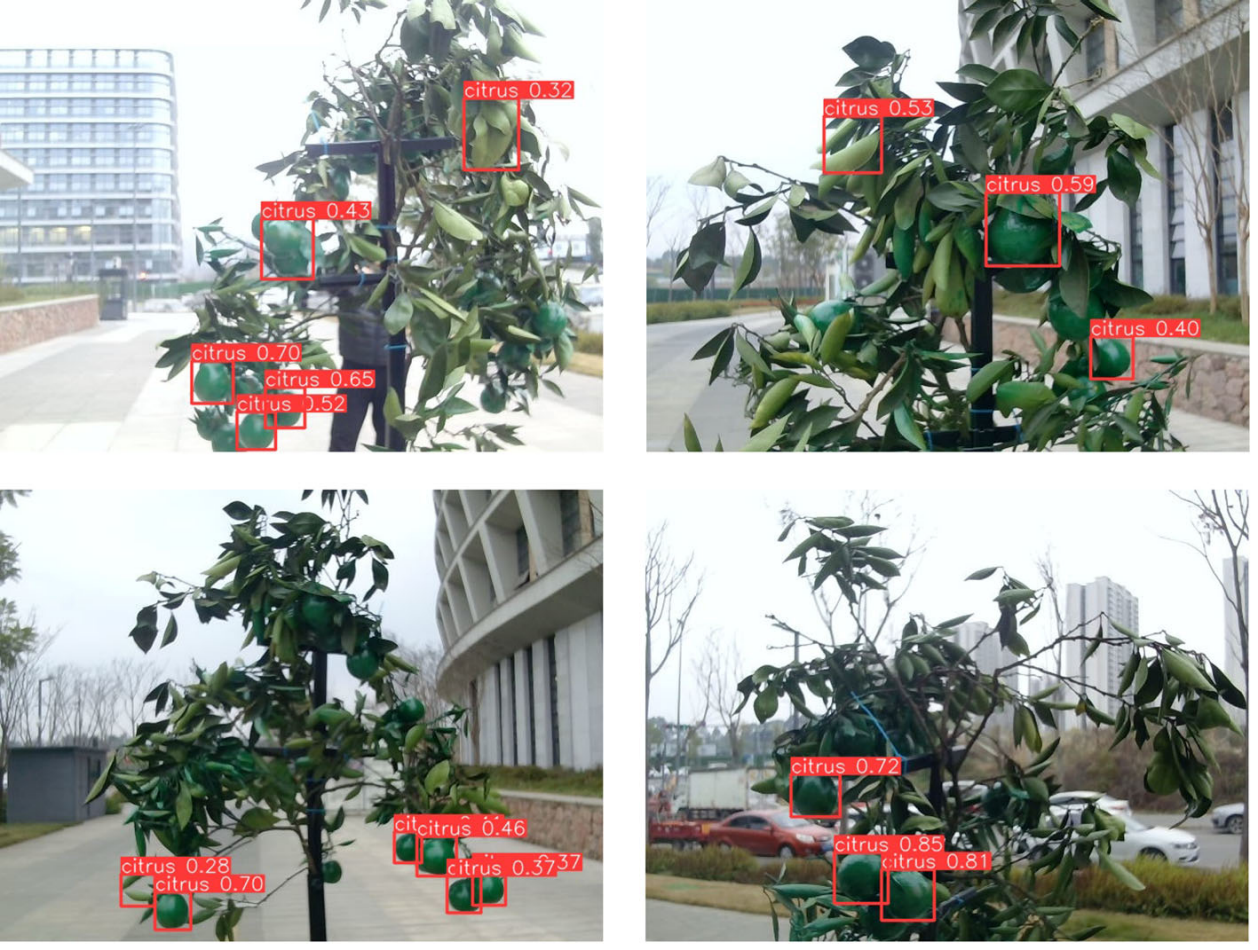
Some test results of the outdoor inspection system.

In order to further verify the reliability in the low-configuration hardware of this model, a citrus young fruit detection system was built outdoors to detect the citrus young fruit model tree as shown in **Supplementary Figure 1**, where the electric trolley was detected around the distance from the citrus young fruit model tree in a circle. In the detection the camera monitors the upper middle and lower citrus young fruits through the regulator respectively, and some of the results are shown in **Figure 14**. As shown in **Figure 14**, the model was able to detect the young citrus fruits in the test environment, with leakage and low detection accuracy during the detection process, which was due to the large differences between the color and background of the citrus in the test environment and the images in the test set of this paper, but it also proved that the model has a certain degree of resistance to interference, and is able to operate in inexpensive equipment.

## 4 Conclusion

In this paper, we present a novel method for citrus young fruit detection, termed YCCB-YOLO. This approach employs a lightweight P-Block to construct both the detection backbone and detection head, integrates multiple attention mechanisms, adopts the SimSPPF-LSKA feature pyramid, and utilizes the Adam optimization function. In the test set of the model of this paper, the P was 91.79%, the R The experimental results indicate that the model proposed in this study maintains a lightweight structure while ensuring detection accuracy. The introduction of the P-Block structure contributes significantly to the model’s lightweight design. Moreover, the integration of various attention mechanisms enhances the model’s focus on critical areas within images. The SimSPPF-LSKA feature pyramid structure facilitates multi-dimensional feature fusion, thereby improving target detection accuracy. The incorporation of the Adam optimization function further strengthens the model’s nonlinear representation and feature extraction capabilities, enhancing its robustness and generalizability. Testing this model within a citrus young fruit detection system has confirmed its reliability in low-configuration hardware environments.

Considering other well-known objective evaluation metrics, such as accuracy, recall rate, and mAP, this research not only provides a new solution for the task of citrus young fruit detection but also promises to offer citrus growers and managers a more reliable and efficient tool for young fruit detection, fostering the intelligent development of the citrus industry. However, the dataset used in this study is limited, posing a potential risk of overfitting. Despite the model’s exceptional performance, it still faces challenges in dealing with the complexities of real-world operational environments. Future research will focus on expanding and enhancing the dataset and optimizing the model structure to further improve the model’s performance and practicality.

We believe that the contributions of this study lie not only in proposing a novel method for citrus young fruit detection but also in demonstrating the vast potential of deep learning applications in the agricultural sector. By continuously improving and optimizing the model, we anticipate providing more intelligent and efficient solutions for agricultural production, thereby advancing the modernization of agriculture.

## Data availability statement

The original contributions presented in the study are included in the article/supplementary material. Further inquiries can be directed to the corresponding authors.

## Author contributions

GA: Writing – original draft. TZ: Conceptualization, Data curation, Writing – original draft. MW: Formal analysis, Funding acquisition, Writing – review & editing. SY: Funding acquisition, Resources, Writing – review & editing. RL: Investigation, Methodology, Writing – review & editing. FY: Project administration, Resources, Software, Writing – original draft. QJ: Methodology, Project administration, Supervision, Writing – review & editing. XL: Supervision, Visualization, Writing – review & editing.
