# Supplementary figures and images for: Fruits hidden by green: an improved YOLOV8n for detection of young citrus in lush citrus trees
Source: Front Plant Sci. 2024 Apr 10;15:1375118. doi: 10.3389/fpls.2024.1375118 (PMC11039839; doi:10.3389/fpls.2024.1375118)

Figure 1 Outdoor citrus detection system


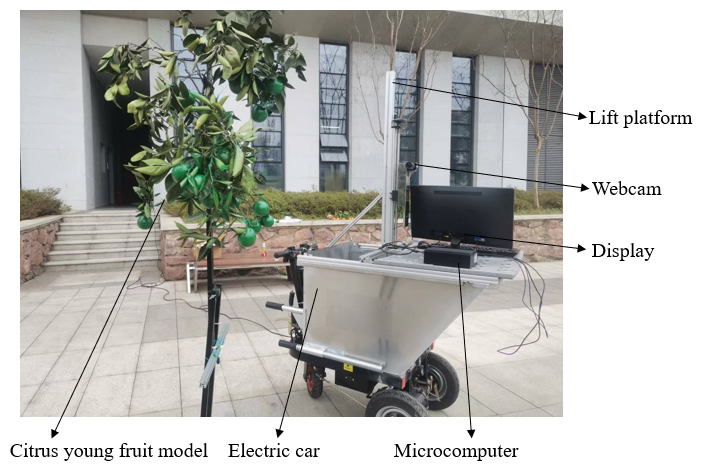

Supplement: Supplementary file 1 [file DataSheet_1.docx]
